# Study of the Differential Consequences of Neglect and Poverty on Adaptive and Maladaptive Behavior in Children

**DOI:** 10.3390/ijerph17030739

**Published:** 2020-01-23

**Authors:** Carlos Herruzo, Antonio Raya Trenas, María J. Pino, Javier Herruzo

**Affiliations:** Department of Psychology, University of Córdoba, 14071 Córdoba, Spain; z42hepic@uco.es (C.H.); m02ratra@uco.es (A.R.T.); ed1piosm@uco.es (M.J.P.)

**Keywords:** neglect, poverty, externalizing problems, internalizing problems, adaptive skills, school problems

## Abstract

The consequences of physical neglect on retardation in the development of adaptive behaviors and the increased risk of poor physical and mental health are well documented. As physical neglect is a phenomenon found almost exclusively among socially deprived people, it is important to distinguish the health effects caused by neglect from those caused by poverty. The objective of this study was to compare the effects of poverty and physical neglect on the development of problematic externalizing and internalizing behaviors, adaptive skills, and school problems among school children between the ages of 3 and 12. A group of 157 children were chosen from 28 Andalusian schools and classified in three homogeneous groups. Children in group 1 (*n* = 53) had two target conditions: living in slums (poverty) and suffering from neglect. Children in group 2 (*n* = 52) had one target condition: living in the same slums as the children in group 1, but not suffering from neglect. Group 3 (*n* = 52) consisted of children from other (non-slum) neighborhoods who did not suffer from neglect. Adaptive and maladaptive behaviors were evaluated with the Behavior Assessment System for Children (BASC). Significant differences were found between group 1 and group 2, but there were no important differences between group 2 and group 3. The conclusion was that externalizing and internalizing problems, school problems, and low adaptive skills found in neglected children were associated with neglect rather than with poverty or socially deprived environments.

## 1. Introduction

The failure of a caregiver to provide the food, clothing, shelter, medical care, or supervision required to ensure that a child’s health, safety, and wellbeing are not harmed has been defined as physical neglect [1,2], a subtype of child maltreatment with a global prevalence, according to the meta-analysis recently carried out by Van IJzendoorn, Bakermans-Kranenburg, Coughlan, and Reiiman [3], of 163/1000. Neglect has serious consequences for children’s development and for their physical and mental health [1,4,5,6,7].

Since the work of Egeland, Sroufe, and Erickson [8], and despite the difficulty of studying pure typologies [9,10], a great amount of literature has accumulated about the differential consequences of maltreatment subtypes. Neglect affects children’s social, behavioral, and cognitive development [11,12,13,14] and can even alter their brain development and physiology, increasing the risk of poor physical and mental health [9,15]. In the field of social behavior, difficulties have been reported in social interaction, with distorted patterns of interaction with caregivers and peers, fewer social skills such as empathy, poorer interpersonal relationships, and changes in emotional behavior [16,17]. Cognition is also affected, with problems such as attention deficit, difficulties in communication and expressive skills, lower academic achievement, and school maladjustment [12,18].

Moreover, in comparison with other typologies, neglected children have more serious cognitive deficits and socialization problems than abused children. They also appear to display behavior patterns that are more internalizing than externalizing [12,19,20,21], as has been reported by both parents and teachers [22,23]. Kotch et al. [24] noted that externalizing behavior problems may later evolve into aggressive and criminal behavior [25], especially when it occurs before the age of five [24]. In addition to that, the severity of physical neglect is particularly important during the preschool period, where it has been associated with internalizing symptomatology and withdrawn behavior [26], problems which tend later to worsen [27]. However, because these effects depend partly on the culture and the welfare system of each country, there is no full consensus.

Grogan-Kaylor, Ruffolo, Ortega, and Clarke [28] found that although all children who suffered physical abuse, neglect or sexual abuse had higher levels of externalizing problems than non-abused children, the neglect group stood out for its higher number of problems of an internalizing nature. This contrasted with the study by Mills, Scott, Alati, O’Callaghan, Najman, and Strathearn [29]. Other results are contradictory. Those of De Paul and Arruabarrena [30], for example, found that children who suffered physical neglect scored higher in externalizing behaviors such as aggressiveness, hyperactivity, and distraction than those who were physically abused, who had a higher prevalence of internalizing behaviors such as anxiety, obsessive-compulsive behaviors, inhibition, unpopularity and self-destruction.

Nevertheless, as physical neglect is a phenomenon found almost exclusively among socially deprived people (of low financial status), it is important to distinguish aspects related to neglect from those related to poverty. This difference is crucial when designing interventions, insofar that it tells us whether we should focus on providing more resources to reduce poverty or invest in the training of caregivers to take full advantage of the resources they have already received.

The two key factors in situations of neglect, as identified both by Barnett, Manly, and Cicchetti [31] and by English, Thompson, Graham, and Briggs [32], are (a) the responsible adult’s inability to meet the child’s basic physical needs (food, clothing, medical care and shelter) and (b) lack of supervision. In the case of poor families, neglect would occur, for example, if parents or caregivers did not request help in meeting those needs when community resources, such as food banks or shelters, were available (probably due to ignorance or not knowing how to ask for help). Lack of supervision occurs when, for example, the caregiver fails to ensure that the child is not involved in potentially harmful activities or to adequately protect the child from dangerous people [33]. The question, then, is whether the consequences of such situations are associated with poverty or with neglect itself?

Poverty correlates highly with neglect. Its harmful consequences, widely identified in many literature reviews, can be observed from the earliest stages of children’s lives [3,27,34,35,36,37,38,39,40]. Children who live in poor families manifest more behavioral and emotional problems [41]. Low socio-economic status has been associated with higher externalizing behavior problems over time from kindergarten to adolescence [42], with impaired cognitive functioning [43] and with poorer mental health [44,45,46,47,48]. Neglectful families generally have a lower socio-economic status than abusive families [49]. Many studies have demonstrated that children’s home environments and their parents’ emotional wellbeing mediate the relationship between low family income and their emotional and behavioral problems [50,51,52,53,54,55]. However, most studies have focused on studying poverty as a risk factor for child abuse and neglect and vice versa, but the differential role of these variables in the harmful consequences has received very little attention. Jonson-Reid et al. [56], for example, in their systematic review of longitudinal research into experiences of child maltreatment and economic outcomes in adulthood found (despite an extremely limited evidence base) that neglect had a consistent relationship with several long-term economic outcomes such as reduced income, unemployment, lower levels of professional skills, and fewer assets. As Van IJzendoorn et al. [3] recently asserted in a meta-analysis of 28 studies into neglect, socio-economic status is a predictor of elevated risk for child maltreatment. According to Mulder, Kuiper, Van Der Put, Stams, and Assink [57] and Euser, Alink, Tharner, Van IJzendoorn, and Bakermans-Kranenburg [58], children from families with a low educational level, and immigrant families and children with unemployed parents have a significantly higher risk of becoming victims of maltreatment. Maguire and Font [59] studied the relationship between individual and neighborhood levels of poverty in more than 1000 families and found that, whereas moving a non-poor family from a high-poverty neighborhood to a low-poverty neighborhood may reduce the risk of some forms of maltreatment, moving poor families away from high-poverty neighborhoods may have little effect on maltreatment, suggesting the importance of interactions inside the family environment as determinant factors in the results. McLeigh, McDonell, and Lavenda [60] found that social cohesion (mutual trust and shared expectations among neighbors) mediated the association between neighborhood-level poverty and abuse rates but not neglect rates. From a global perspective, economic factors are consistently related to neglect [38], both factors being a major risk for externalizing behavioral problems in the long term. On the other hand, neglect has been linked with internalizing problems in the short-term and with externalizing problems later on [61]. However, it is not yet known whether poverty affects neglect or vice versa, or whether they are independent, interacting factors [40].

In this regard, both Schumaker [62] and Chapple and Vaske [63] argue that it is necessary to discriminate the consequences of poverty on development during childhood and adolescence from those attributable to child abandonment, because poverty alone cannot explain the results of their studies; and yet, as noted by Van IJzendoorn and others [3], researchers have tended to focus almost exclusively on poverty as a risk factor for abandonment or abuse. It is therefore necessary to conduct studies to distinguish the consequences of poverty from those of neglect itself, because very little research has been carried out in this area to date. The objective of this work was to study the differential consequences of neglect and poverty on internalizing and externalizing behavior problems, school problems, and adaptive skills in children. To do so, we compared a group of neglected children living in poor neighborhoods with a control group of the same social class (poverty but non-neglect), and with another control group of children from a different socio-economic background (neither poverty nor neglect). The factorial design was not completed with a fourth group (neglect and not poverty) because neglect is so difficult to find among non-poor people.

## 2. Materials and Methods

### 2.1. Participants

This study involved 157 minors from the cities of Cordoba and Jaen (Andalusia, Spain). They were divided into three groups: group 1 (G1) comprised 54 physically neglected children from marginalized backgrounds, group 2 (G2) comprised 51 children not physically neglected or otherwise abused from the same social background as the G1 group, and group 3 (G3) comprised 52 children from other, non-marginalized backgrounds who were not physically neglected or otherwise abused.

The children in G1 were chosen at random from 187 families identified by the Social Services Family Support Teams as having neglected children. Each child had to meet two requirements: to be in a situation of physical neglect, and to be of preschool or school age (between 3 and 12 years), due to the special relevance of this age group for this type of research [24]. Two members of the research team and two social workers selected children randomly from Social Services Records, ensuring that each one met the inclusion criteria, to produce a total sample in which 33% were children aged between 3 and 5, 33% were between 6 and 8, and 33% were between 9 and 12. The group was made up of 31 boys and 23 girls, with an average age of 7.20 (SD = 2.67). The group members came from depressed, peripheral areas of both cities, where socio-economic and educational levels are usually quite low. The team then contacted the 18 schools where these children were schooled, requesting permission to conduct the research. The children in the G2 group were chosen at random, in collaboration with the teacher of each G1 child, among children from other families living in the same area with low socioeconomic levels and with children at the same school, ensuring that the G2 subjects did not suffer physical neglect or any other type of maltreatment. To this end, the teachers filled out the “School Child Abuse Risk Notification Form” from the Andalusian Child Maltreatment Information System [64], an instrument used by state schools in Andalusia, and the Social Services then corroborated that the children in question were not subject to neglect or maltreatment. Each child was matched one by one with a G1 counterpart of the same age, socio-economic level, and sex. In this group, made up of 26 boys and 25 girls, the average age was 7.39 (SD = 2.68). The children in the G3 group were chosen at random from 10 state schools in non-marginalized areas of both cities, with the collaboration of the schools’ principals. Again, they were matched one by one with members of the other groups of the same age and sex, care being taken (as with the G2 group) to ensure that none of those included in this group suffered physical neglect or any other type of maltreatment. The cultural and economic levels of these children’s families had to be medium or high, with no record either of physical neglect or any other form of maltreatment. This group was made up of 27 boys and 25 girls, with an average age of 7.02 years (*SD* = 2.70).

Homoscedasticity tests applied to check the equivalence of the three groups with respect to sex and age did not yield significant results, with χ^2^ = 0.51 (*p* > 0.05) for sex and *F* = 0.25 (*p* > 0.05) for age. These data confirmed the equivalence between the three groups.

### 2.2. Instruments

To compile information, the following instruments were used:

A sociodemographic questionnaire, to collect data such as the child’s date of birth, age, school year, sex, and school. In this questionnaire, the teacher also identified the group to which the child belonged (G1, G2, or G3), as indicated above in the section on participants. The teacher also filled out the “School Child Abuse Risk Notification Form” from the Andalusian Child Maltreatment Information System, and this was processed by the Social Services in order to corroborate whether or not the children suffered neglect. This form is part of the official maltreatment notification procedure in Andalusia.

A Spanish language adaptation of the “Behavior Assessment System for Children” (BASC) [65]. The purpose of this system is to evaluate a wide range of pathological and adaptive dimensions using different sources of information (parents, teachers, and children) and different methods (questionnaires, developmental history, and observation). In this case, questionnaires for teachers were used. These questionnaires, which are divided into three levels according to age (3–6, 6–12, 12–18), have an internal consistency index of 0.70. Test–retest correlation (three month interval) was 0.85, 0.88, and 0.70 for the three levels of the questionnaire the teachers completed.

This study used the main composite dimensions of the BASC, together with their different scales. The composite dimensions are externalizing problems (aggression, hyperactivity, and behavior problems), internalizing problems (depression, anxiety, and somatization), school problems (attention problems and learning problems), and adaptive skills (social skills, leadership, and adaptation). Atypicality, withdrawal, and study skills, which are not included in the system’s composite dimensions, were also assessed.

The scores obtained on any of the scales are transformed into T scores, which indicate the extent to which a particular score differs from the control group mean, thereby enabling comparisons to be made between subjects of different ages. These T scores can vary between 0 and 100, with a mean value of 50 and SD of 10. On the basis of the T scores, different levels are established: scores below 30 are considered very low, under 40—low, between 40 and 60—intermediate, over 60—at risk, and over 70—clinically significant.

### 2.3. Procedure

We began the study by contacting the Social Services of the corresponding town councils and the principals of all the schools involved, in order to explain the objectives of the research and to request a list of families identified by the Family Support Teams. A group of families with children between 3 and 12 years of age in situations of physical neglect was chosen at random from a total of 187 families identified as high-risk, in the way described in Section 2.1 and in collaboration with the Social Services and teachers. The families were contacted in order to obtain the necessary consent to carry out the research. Once consent had been obtained from the families and professionals in the schools, the questionnaires were filled in by the children’s teachers in time set aside outside ordinary class time, with the aim of making the atmosphere as relaxed as possible, avoiding distractions and promoting concentration. Neither tutors nor parents were aware of the objectives set for this research, or of the children’s division into the three groups explained in the section on participants. The confidentiality of the participants was at all times guaranteed, and their data were used solely for scientific purposes within the context of this study.

All subjects were treated in accordance with the ethical rules of the American Psychological Association and gave their informed consent. The study was conducted in accordance with the Declaration of Helsinki, and the protocol was approved by the Ethics Committee at the University of Córdoba (191119).

### 2.4. Data Analysis

The research objectives were addressed using an ex-post-facto prospective design with three comparison groups. The two independent variables (IV) available in this study were therefore:

IV 1: Physical neglect as a subtype of child maltreatment, establishing two levels: yes/no.

IV 2: Family socio-cultural context, again establishing two levels: marginalized/non-marginalized.

Only three groups were formed because complete factorization between the two IVs was not possible given that neglect is so difficult to find in non-marginalized sociocultural contexts.

The dependent variables were the T scores obtained for the different externalizing and internalizing problems, school problems, and adaptive skills measured in the teachers’ version of the BASC mentioned above. In order to be able to disassociate the effects of physical neglect from those typically experienced in situations of marginalization, the design used analysis of variance (ANOVA) to explore differences among groups and further planned comparisons in cases in which significant differences were found. In order to minimize possible type I errors, only the two planned post-hoc comparisons related to the research objectives were carried out (G1 vs. G2 and G2 vs. G3), using a level of significance of 0.025 [66]. For determining the optimal post-hoc test to use in each case, the Levene’s variance homogeneity test was used. For comparisons where group variances were homogeneous, the HSD (Honestly-significant-difference) Tukey test was used, whereas for comparisons where groups had significantly different variances, Dunnett’s T3 test was used [66].

For all comparisons, the size of the effect was calculated using the coefficient η². For this purpose, we considered the classification made by Cohen [67], according to which a value of η² lower than 0.1 would be low, between 0.1 and 0.25 would be moderate, and between 0.25 and 0.4 would be high.

## 3. Results

This study aimed to analyze the differential consequences of neglect and poverty on internalizing and externalizing behavior problems, school problems, and adaptive skills in children. The strategy adopted to do this was to compare a group of neglected children with a control group of non-neglected children of the same social class, and with another control group of children from a different socio-economic background. Several ANOVAs were carried out, providing us with information on the possible existence of differences between the three groups and between which groups differences really did exist.

**Figure 1:**
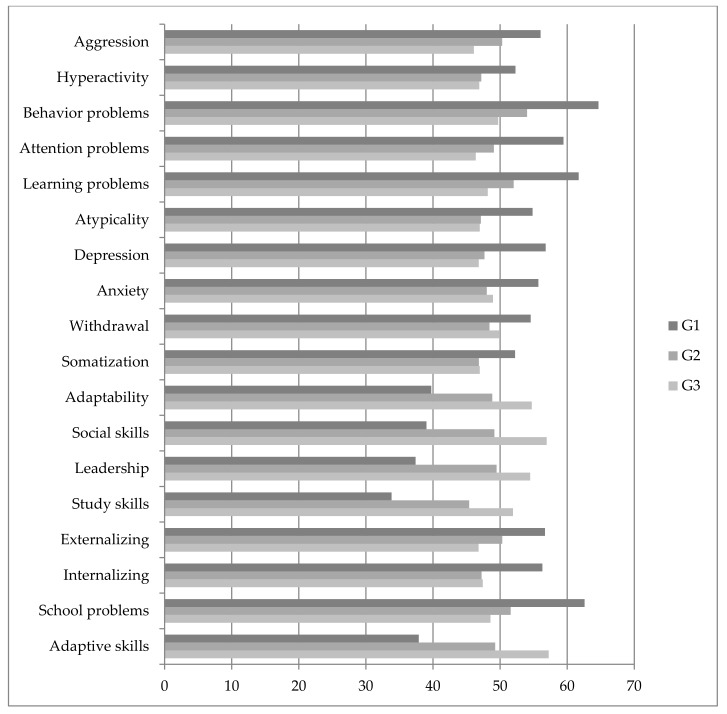
Average scores obtained by the three groups for the different dependent variables. All comparisons were significant for alpha = 0.05.

Figure 1 shows the average T scores obtained by the three groups for the different dependent variables studied. Table 1 (captioned ANOVA) shows the results of ANOVA (*F*, *p*) and effect size (η²) for each dependent variable. Significant differences were obtained in all the variables analyzed: aggression, hyperactivity, behavior problems, attention problems, learning problems, atypicality, depression, anxiety, withdrawal, somatization, adaptability, social skills, leadership, and study skills. Differences were also found in the composite dimensions analyzed in the study: externalizing problems, internalizing problems, school problems, and adaptive skills. The effect size (η²) was moderate for several variables (such as aggression, attention problems, learning problems, atypicality, depression, adaptability, externalizing problems, internalizing problems, and school problems), with η² values between 0.1 and 0.25, whereas a higher effect was obtained for variables such as social skills, leadership, study skills, and adaptive skills, with values higher than 0.25 (according to the levels set by Cohen [67]).

As the ANOVA found significant differences in all dependent variables, planned comparisons were analyzed to explore differences among groups. The four right-hand columns in Table 1 show *p*- and η² values for the G1 vs. G2 and G2 vs. G3 comparisons (missing η² values correspond to non-significant differences in *p*-values). Significant differences were obtained between group G1 and group G2 for most of the variables studied. Only in the cases of aggression, somatization, and externalizing problems did the G1 group not differ significantly from the G2. In contrast, there were no significant differences between G2 and G3 except for in social skills and adaptability skills. As can be seen in Table 1, the effect size (η²) obtained was high in all the post-hoc comparisons that were significant, highlighting the differences between G1 and G2.

## 4. Discussion

This study aimed to analyze the differential consequences of neglect and poverty on internalizing and externalizing behavior problems, school problems, and adaptive skills in children. For this purpose, three carefully selected groups of children were compared. The main differentiating elements in these sample groups were, on one hand, neglect, and on the other, a marginalized background. The results obtained suggest that suffering neglect is the main risk factor for a wide spectrum of adaptation problems, including internalizing problems, school problems, and lack of adaptive skills and some externalizing problems. The principal differentiating element, and thus probably the main determinant of the presence of the adaptation problems studied, can therefore be said to be the presence of physical neglect rather than a child’s socio-economically marginalized background, because the results show differences between neglected and non-neglected children from marginalized backgrounds but no differences between children from marginalized and non-marginalized backgrounds.

This study could be a small step beyond the assertion of Van IJzendoorn et al. [3] that a low socio-economic level is a risk factor for the possible presence of neglect. Following the suggestions made by Schumaker [62] and Chapple and Vaske [63], it sheds some light on the controversy inasmuch that it is important to distinguish between poverty and neglect as predictors of adaptation problems. As the aforementioned authors assert, poverty alone cannot explain the results of the studies. Our results help reinforce this idea. Although several authors, such as Bradley and Corwyn [41] or Landsford et al. [42], have focused on how a child’s socio-economic situation can affect adaptation problems, it is important to consider that the worst levels of adaptation in children may actually to a large extent be explained by neglect alone [62,63]. This is particularly the case for attention problems and learning problems, but it is also true of some internalizing factors such as depression, where significant differences were found between the neglect group and the marginalized background group with a moderate effect size. To the extent that these problems can be observed in a school context, they can be said to cause children to fail and achieve less academic and professional success, as well as perpetuating marginality. In the case of social and adaptive skills, however, there are also some quite prominent differences between children in marginalized contexts and children in favored contexts. These results are congruent considering that context seems also to play an essential role in the development of certain adaptive skills. Furthermore, the comparisons carried out for these adaptive factors were those with the largest effect sizes, and so the idea of context as an important element in the development of these skills, together with the effects of neglect, thus represents a major area of study for future research aimed at developing models capable of explaining the presence or absence of these skills so vital to a child’s social and personal development.

Ultimately, although we agree with other previous studies that neglect and poverty are present simultaneously in many cases [3,27,34,35,36,37,38,39,40], our study suggests that it is not a low socio-economic level that causes the main adjustment problems in children, but rather neglect by their parents or caregivers who, despite the existence (sometimes) of resources in the community for alleviating the effects of poverty, lack the skills to take advantage of them. This is one of the main practical implications of the results our study—the need to promote teaching and training to help negligent parents use community resources properly.

Regarding types of maltreatment, our results do not reflect the differences reported by authors such as Grogan-Kaylor et al. [28] and Mills et al. [29], who suggest a closer relationship between externalizing problems and physical maltreatment, thus linking internalizing problems with neglect more directly. In the present study, although the differences are quite large with regard to internalizing problems, considerable differences were also found in externalizing problems, school problems, and adaptive skills.

Given that there are some weaknesses in the study, such as the use of questionnaires to evaluate children’s behavior or the smallness of the effect obtained for some variables, the thoroughness with which the three groups of participants were selected and the use of teachers as a source of information on the children’s behavior made it possible to avoid possible biases that would have distorted the results of the study. Using parents as a source of information would have entailed a high risk of obtaining responses conditioned by social desirability, whereas not having been exhaustive in the selection of the three groups would also have distorted the results, as has occurred in other studies where the effect of neglect on adaptation problems could be confused with that of belonging to a marginal environment. This is the great strength of these results. Despite the fact that children from a marginal context showed worse results than those from the favored context, the most severe differences were found between the group of neglected children and both of the other groups, thus strongly indicating that the main differentiating element is really neglect itself.

As was mentioned earlier, and in accordance with the findings of Barnett et al. [31] and English et al. [32], the two key factors in neglect are (a) the inability of the responsible adult to provide for the child’s basic physical needs, and (b) lack of supervision. These shortcomings are, precisely, the main key to interpreting the present results, which are concurrent with research about parental styles and behavior problems that a lack of supervision or limits inside the family predicts externalizing behavior problems [68,69].

## 5. Conclusions

To conclude, the question we asked in the introduction to this study was whether the consequences of neglect are associated with poverty or with neglect itself. The present results threw some light on the issue, indicating higher levels of maladaptive behavior among children who suffer from physical neglect, and no significant differences between non-neglected children, regardless of their socio-economic background. It is therefore still necessary to study both factors separately because, although there seems to be a high level of interaction between the two, neglect is the most determinant element. It would be necessary to place more emphasis and attention on adopting measures aimed at minimizing the effects of neglect on children, working with families and institutions to make the best use of the available resources, in order to ensure children’s welfare and to safeguard their fundamental rights.

## Figures and Tables

**Table 1:** Results of ANOVA, effect size, and post-hoc comparisons.

Variable	ANOVA			G1–G2		G2–G3	
	*F*	*p*	η²	*p*	η²	*p*	η²
Aggression	9.76	<0.001	0.115	0.070		0.160	
Hyperactivity	4.86	0.009	0.060	0.027		0.988	
Behavior problems	7.37	<0.001	0.041	0.022	0.318	0.527	
Attention problems	23.05	<0.001	0.233	<0.001	0.445	0.394	
Learning problems	10.21	<0.001	0.185	0.006	0.358	0.435	
Atypicality	9.63	<0.001	0.114	0.004	0.314	>0.999	
Depression	12.42	<0.001	0.142	<0.001	0.355	0.964	
Anxiety	7.63	<0.001	0.093	0.003	0.324	0.949	
Withdrawal	4.19	0.017	0.053	0.018	0.265	0.798	
Somatization	4.97	0.008	0.063	0.036	0.244	>0.999	
Adaptability	22.51	<0.001	0.231	<0.001	0.357	0.029	
Social skills	36.04	<0.001	0.325	<0.001	0.429	<0.001	0.319
Leadership	25.02	<0.001	0.360	<0.001	0.519	0.208	
Study skills	20.30	<0.001	0.313	<0.001	0.467	0.141	
Externalizing	9.26	<0.001	0.111	0.029		0.267	
Internalizing	12.25	<0.001	0.142	<0.001	0.372	>0.999	
School problems	11.82	<0.001	0.208	<0.001	0.410	0.592	
Adaptive skills	36.40	<0.001	0.328	<0.001	0.433	0.002	0.314
